# Psychopathology, Dissociation and Somatic Symptoms in Adolescents Who Were Exposed to Traumatic Experiences

**DOI:** 10.3389/fpsyg.2018.02390

**Published:** 2018-12-04

**Authors:** Chiara Luoni, Massimo Agosti, Sara Crugnola, Giorgio Rossi, Cristiano Termine

**Affiliations:** ^1^Child Neuropsychiatry Unit, Mother and Child Department, Del Ponte Hospital, Azienda Socio Sanitaria Territoriale (ASST) Sette Laghi, Varese, Italy; ^2^Neonatology Unit, Mother and Child Department, Del Ponte Hospital, Azienda Socio Sanitaria Territoriale (ASST) Sette Laghi, Varese, Italy; ^3^Department of Medicine and Surgery, University of Insubria, Varese, Italy

**Keywords:** complex trauma, dissociation, somatic symptoms, adolescents, PTSD

## Abstract

**Background:** The direct and long-term effects of children’s exposure to traumatic events can be seen in a complex continuum, based first of all on the type of trauma. Children’s reactions to trauma may have different manifestations from the clinical picture of the PTSD, exhibiting dissociative, somatic, depressive or anxiety symptoms, and/or disruptiveness.

**Aim:** we conducted a cross-sectional study in a psychiatric patients sample to determine the extent to which complex trauma history is associated with disease-related characteristics (diagnosis, dissociative symptoms, somatic symptomatology, impairment degree).

**Methods:** We have enrolled 107 subjects, aged between 12 and 18 years, who consecutively referred for a psychiatric evaluation to the Child Neuropsychiatry Unit of the Del Ponte Hospital in Varese. All subjects underwent a clinical evaluation performed by infantile neuropsychiatrists. The battery of tests that was administered to patients included CGI and CGAS (filled out by the clinician), CBCL (filled out by parents), MMPI-A and TSSC-A (filled out by patients), and Wechsler scale.

**Results:** We found out that 35.5% of subjects had a mood disorder, 23.4% a personality disorder, 13.1% a psychotic disorder, 20.6% a post-traumatic stress disorder, while 26.2% were classified as other diagnostic categories (more frequently ADHD, DOP and conduct disorders). 58.9% of patients had at least one comorbidity. 33.6% of subjects also experienced a complex trauma. In multivariate logistic regression analyses, subgroup fellows were collapsed to compare the single trauma and no trauma versus complex trauma group. Gender, age and affective disorders were generally unrelated to subjects’, clinicians’, and parents’ scores. About subjects’ self-assessment (MMPI-A Structural Summary Factors), complex trauma history was a statistically significant contributor to high scores on the Immaturity, Health Concerns, Familial Alienation and Psychoticism Factors, followed by presence of dissociative symptoms (except for Familial Alienation factor). Presence of dissociative symptoms, personality and psychotic disorder diagnosis was related to higher clinician impairment scores (CGI-S > 4).

**Conclusion:** These results reinforce available evidence that in trauma-exposed adolescents, the full burden of trauma, including other psychiatric diagnosis than PTSD (such as affective, personality, and psychotic disorders), dissociative and somatic symptomatology, is substantial and needs appropriate assessment and therapeutic interventions.

## Introduction

Psychological trauma is the individual experience of an event or prolonged conditions in which the individual’s ability to integrate his/her emotional experience is overwhelmed or the individual experiences a threat to life, bodily integrity or sanity, intense fear, terror, and helplessness. Therefore, a traumatic event or situation creates psychological trauma when it overcomes the individual’s ability to cope and leaves the person fearing death, destruction, mutilation or psychosis. The individual may feel emotionally, cognitively and physically overwhelmed. Many events are included in the definition of trauma such as accidents, natural disasters, crimes, surgeries, deaths, and other violent events; it also includes responses to chronic or repetitive experiences such as child abuse, neglect, combat, urban violence, concentration camps, battering relationships and enduring deprivation (Pearlman and Saakvitne, 1995).

The stressors causing post-traumatic stress disorder, included in Criterion A of the PTSD in DSM 5 (American Psychiatric Association [APA], 2013), consists in experiencing a serious threat to life or physical integrity, witnessing serious threat, harm or death, learning about violent threat or harm to a close friend or relative, sudden destruction of home or community; the range of stressors reported in children includes kidnapping and hostage situations, exposure to violence, witnessing rape, murder or suicide behavior, sexual or physical abuse, severe accidental injury (including burns, hit-and-run accidents, animal bites, toxic exposures), life-threatening illness of life-endangering medical procedures, severe automobile, railroad, airplane, ship or boating accidents, natural, or human disasters.

However, children’s reactions to trauma may have different manifestations from the clinical picture of the PTSD described in the DSM-5; for example after experiencing a trauma children may present somatic, depressive or anxiety symptoms, inattention/hyperactivity symptoms, and/or disruptiveness.

The direct and long-term consequences of children’s exposure to traumatic experiences can be seen in a complex continuum, based first of all on the characteristics of trauma, in particular the severity, the duration of exposure and its precocity (English et al., 2005; Jonkman et al., 2013; Hodgdon et al., 2018). Complex traumatic exposure refers to the relevant experience to multiple traumatic events that occur in the primary care system, namely in that specific social environment that assumes to be the main source of stability and security for the child’s life. Generally concurrent or sequential experiences include mistreatment, emotional abuse, neglect, violence, sexual and physical abuse, that are chronic and occur in early childhood (van der Kolk et al., 2005; Sachser et al., 2017). People who were exposed to early onset, numerous, prolonged, and sometimes highly aggressive traumatic events, frequently due to other people, often associated with a significant amount of stigma or discredit, may be more susceptible to stress effects. Recurrent experiences of hurt and/or rejection by familiar persons, and the consequent failure to achieve adequate skills for the age, can lead children to feel themselves faulty, helpless, imperfect, and hateful. Children often expect to be rejected or denigrated by other people and feel helpless and incapable, therefore they accuse themselves of negative experiences and are unable to call for help and support to others. Thus, the experience of a complex trauma can have a negative impact on the ability of self-regulation and interpersonal relationship, with consequent increased possibility of developing substance abuse, psychiatric or medical disorders, legal, professional, or familiar problems (Cook et al., 2005).

Only a part of children who have experienced trauma develop a post-traumatic stress disorder (Margolin and Vickerman, 2007); some may have some symptoms (flashbacks, intrusive memories, sleep disorders) without reaching all diagnostic criteria. In fact, subsequent reactions to a traumatic event can have different symptom expressions: besides post-traumatic stress disorder, traumatized individuals may develop somatic distress, mood disorders, impairment of cognitive, and interpersonal functioning and dissociative symptoms (Briere and Spinazzola, 2005).

Traumatized children can often develop difficulty in maintaining attention and in executive functions, with an impact on reasoning and abstraction skills. They are more frequently referred for special education services, they often respond less to standard treatments and they rarely reach higher levels of education (Ford et al., 2009).

There can be the onset of dissociative symptoms that are characterized by disconnection and/or discontinuity of the normal integration of conscience, memory, identity, emotion, perception, bodily representation, motor control, and behavior. They can potentially affect all areas of psychological functioning and they can be experienced as (a) unwanted intrusions into consciousness and behavior, associated with the loss of continuity of subjective experience (for example, “positive” dissociative symptoms as fragmentation of identity, depersonalization and derealization) and/or (b) inability to access information or to check mental functions that are generally easily accessible or controllable (for example, “negative” dissociative symptoms as amnesia). Even though dissociative symptoms may operate as a mechanism to decrease the emotional distress related to traumatic events, chronic exposure to traumatic facts can bring to an excessive appeal to the dissociation to defensive purpose, with consequent serious repercussions on the emotional, behavioral and cognitive self-regulation (Greeson et al., 2012).

Traumatic events and their consequences can cause or significantly intensify bodily distress or dysfunction, that are closely related to somatoform symptoms. Children who have experienced trauma are more susceptible to increased rates of somatic symptoms, that can have a negative impact on their functioning and on the quality of life. Besides such symptoms can be often chronic and intense, to such a degree as to require medical investigations, sometimes invasive, to exclude possible organic causes. The diagnostic and therapeutic procedures can be in turn stressful for the child, besides increasing direct medical costs (Kugler et al., 2012).

Early traumatic experiences can have long-term consequences and are associated with an increased risk of psychiatric illness in adulthood, like chronic PTSD, substance abuse, anxiety and mood disorders, eating disorders, and sleep disorders (Arrow, 2004; Chen et al., 2010; Yanos et al., 2010).

In addition, adults with a post-traumatic stress disorder secondary to abuse or mistreatment in childhood, report more frequently than the general population, somatic symptoms and disturbances (Arrow, 2004), such as gastrointestinal disorders, (Paras et al., 2009), chronic pain (Raphael and Widom, 2011), chronic daily headache (Juang and Yang, 2014), migraine (Tietjen et al., 2010), urological symptoms (Chiu et al., 2017).

In view of all these considerations, we conducted a cross-sectional study in a psychiatric patients sample to determine the extent to which complex trauma history is associated with disease-related characteristics (diagnosis, dissociative symptoms, somatic symptomatology, impairment degree).

## Materials and Methods

Over the period 2015–2017, we enrolled outpatients referred for a psychiatric evaluation at the Child and Adolescent Neuropsychiatry Unit at University of Insubria, Varese, according to these criteria: (1) age between 12 and 18 years; (2) absence of intellectual disability; (3) absence of neurological or medical diseases; (4) signature of written informed consent provided by the parents or the legal tutors.

Patients came to the assessment at the request of the parents or of social services (in the case of children placed in communities specialized in abused and mistreated children).

The local Ethics Committee approved the study.

An anamnestic form was filled out for each patient to obtain demographic data, details on personal history, co-morbidities and any clinical symptoms and signs. They were evaluated by neuropsychiatrists with substantial expertise in adolescent psychiatry and they had a DSM-5 validated diagnosis. We considered as “trauma” episodes of sexual abuse, physical violence, abuse or severe neglect, confirmed by the Judicial Authorities (with at least a judgment of first instance born by the perpetrator).

The sample was divided into three groups, based on the personal history: (1) no trauma group (subjects without documented histories of severe trauma and/or abuse); (2) single trauma group (subjects with single traumatic event histories); (3) complex trauma group (subjects with multiple traumatic experiences at an early age within a family context).

After the clinical evaluation, all participants completed a standardized battery including the following psychometric instruments (test and self-report scales).

The *Wechsler Intelligence Scale for Children-IV* (Wechsler, 2010) is used to study intelligence in patients.

The *Minnesota Multiphasic Personality Inventory–Adolescent version* (Butcher et al., 2001): it is a self-report questionnaire consisting of 478 items for administration to people aged between 13 and 18 years. The MMPI-A variables under consideration were the eight factors (General Maladjustment, Immaturity, Disinhibition/Excitatory Potential, Social Discomfort, Health Concerns, Naivete, Familial Alienation, Psychoticism) of the Structural Summary (Archer, 2005).

The *Trauma Symptom Checklist for Children* (form TSCC-A): it is a 44-item self-report scale designed to measure post-traumatic symptoms in children aged between 8 and 16 years, without the sexual concerns scale (included in the form TSCC) (Briere, 2011). In accordance with the recommendations made in the manual, clinicians have also administered the TSCC to 17 years old patients (i.e., Singer et al., 1995; Fricker and Smith, 2001; Finkelhor et al., 2007). Responses on the TSCC-A ranged from 0 (never) to 3 (almost always). Summed scores were converted to T-score according to norms established by gender and age.

The parents or the legal tutors of all subjects completed the *Child Behavior Checklist* (Achenbach, 2001), a parent-reported questionnaire for children and adolescents aged 6–18 years. It consists in 118 items and assesses the child’s internalizing symptoms (anxiety, depression, withdrawal, and somatic complaints) and externalizing behaviors (including thought problems, attention problems, rule breaking, and aggressiveness).

The clinicians filled the *Clinical Global Impressions-Severity of Illness Scale* (Busner and Targum, 2007), a clinician-completed scale extensively used to assess the overall severity of a patient’s psychiatric disease on a Likert Scale ranging from 1 (“not ill”) to 7 (“extremely severe”).

### Statistical Analysis

The Kolmogorov-Smirnov test confirmed the normal distribution of all non-categorical variables; for descriptive statistics, the measures used were means (standard deviations) for continuous variables, and percentage distributions for categorical variables.

The three groups were compared to identify differences in demographic data, disease-related characteristics and psychometric measures, obtained by administered test and questionnaires. Frequency distributions were compared by chi-square test and means by independent samples one-way ANOVA. Next, multivariate analysis (logistic regressions) were conducted in order to determine whether age, gender, psychiatric diagnosis and complex trauma history could be significant contributors to differences in psychometric measures. All statistical analyses were conducted with SPSS Version 17.0 (SPSS Inc.). The level of statistical significance was set at 0.05.

## Results

One hundred and seven subjects, 44 males and 63 females, were enrolled; their demographic and disease-related characteristics are shown in Table 1. Mean age was 15 years (range 12–18) and the vast majority of subjects (67.3%) had a low socio-economic status. 21.5% were migrant and 9.3% were adoptive children; 25 patients (23.4%) lived in out-of-home placements. Almost one third (29%) had a scholastic failure and 35.5% had learning disabilities. Psychiatric comorbidities were present in 59% of subjects: the majority of subjects (35.5) had affective disorders, 26.2% had ADHD or conduct disorder, 23.4% had personality disorders, 20.6% post-traumatic stress disorders, and 13.1% psychotic disorders. Furthermore 28% had dissociative symptoms. 35.5% took drug therapy at the time of the evaluation. Self-harm was frequent (31.8%) and 15% had at least one suicide attempt. Substance and alcol abuse was detected in 17.8 and 6.5% respectively.

**Table 1:** Demographic data and disease-related characteristics of the total sample.

Characteristics	Total sample 107 (100)	No trauma 59 (55.2)	Single trauma 12 (11.2)	Complex trauma 36 (33.6)	*p*-value
**Gender**					
Male	44 (41.1)	30 (50.8)	4 (33.3)	10 (27.8)	0.072^∗^
Female	63 (58.9)	29 (49.2)	8 (66.7)	26 (72.2)	
Mean age, years (SD)	15.09 (1.76)	15.35 (1.76)	14.94 (1.86)	14.71 (1.71)	0.216°
Low socio-economic status	70 (67.3)	35 (62.5)	9 (75.0)	26 (72.2)	0.520^∗^
Adoptive child	10 (9.3)	1 (1.7)	1 (8.3)	8 (22.2)	**0.004^∗^**
Migrant	23 (21.5)	7 (11.9)	3 (25.0)	13 (36.1)	**0.019^∗^**
**Ethnicity**					
White	84 (78.5)	54 (91.5)	8 (66.7)	22 (61.1)	**0.010^∗^**
Hispanic	15 (14.0)	2 (3.4)	2 (16.7)	11 (30.6)	
African	5 (4.7)	2 (3.4)	1 (8.3)	2 (5.6)	
Asian	3 (2.8)	1 (1.7)	1 (8.3)	1 (2.7)	
Out-of-home placement	25 (23.4)	2 (3.4)	1 (8.3)	22 (61.1)	**<0.001^∗^**
Scholastic failure	31 (29.0)	17 (28.8)	6 (50.0)	8 (22.2)	0.185^∗^
Learning disability	38 (35.5)	22 (37.3)	5 (41.7)	11 (30.6)	0.717^∗^
Dissociative symptoms	30 (28.0)	8 (13.6)	4 (33.3)	18 (50.0)	**0.001^∗^**
Affective disorder	38 (35.5)	22 (37.3)	5 (41.7)	11 (30.6)	0.717^∗^
Personality disorder	25 (23.4)	10 (16.9)	4 (33.3)	11 (30.6)	0.216^∗^
Psychotic disorder	14 (13.1)	9 (15.3)	1 (8.3)	4 (11.1)	0.739^∗^
Post-traumatic stress disorder	22 (20.6)	0 (0.0)	1 (8.3)	21 (58.3)	**<0.001^∗^**
ADHD/Conduct disorder	28 (26.2)	22 (37.2)	4 (33.3)	2 (5.5)	**<0.001^∗^**
Comorbidity	63 (58.9)	31 (52.5)	7 (58.3)	25 (69.4)	0.267^∗^
Substance abuse	19 (17.8)	9 (15.3)	2 (16.7)	8 (22.2)	0.679^∗^
Alcol abuse	7 (6.5)	3 (5.1)	1 (8.3)	3 (8.3)	0.796^∗^
Self-harm	34 (31.8)	13 (22.0)	6 (50.0)	15 (41.7)	**0.049^∗^**
Suicide attempt	16 (15.0)	5 (8.5)	3 (25.0)	8 (22.2)	0.111^∗^
Pharmacotherapy	38 (35.5)	19 (32.2)	3 (25.0)	16 (44.4)	0.584^∗^
**Abuse History**					
Physical abuse	36 (33.6)	0 (0.0)	6 (50.0)	30 (83.3)	**<0.001^∗^**
Sexual abuse	32 (29.9)	0 (0.0)	6 (50.0)	26 (71.2)	
Neglect	23 (21.5)	0 (0.0)	3 (25.0)	20 (55.6)	

*Differences between the three groups, assessed by Chi-Square (^∗^) and One-Way ANOVA (°). Reported data are number of patients, with percentages shown in brackets, unless specified otherwise. Bolded values were statistically significant p-values.*

55.2% of patients had no histories of trauma and/or abuse (”No trauma group”); 11.2% had a single traumatic event (”Single trauma group”); 33.6% had multiple traumatic experiences (such as physical or sexual abuse, emotional or physical neglect) at an early age within a family context (”Complex trauma group”). There were no significant differences between the three groups in terms of gender, mean age, socio-economic status and the vast majority of disease-related characteristics (Table 1). In particular, the three groups did not differ in likelihood of having affective, personality or psychotic disorders. Not surprisingly, 95.5% of patients with post-traumatic stress disorder were in the Complex Trauma group, but it is noteworthy that almost half (41.7%) of the subjects in this group did not have a post-traumatic stress disorder. Dissociative symptoms and self-harm were more frequent in patients with simple or complex trauma.

One-way ANOVAs showed, as expected, that the complex trauma group scored higher than the ”no trauma” and ”single trauma” groups on almost all TSSC-A scales (Anxiety, Depression, Anger, Post-traumatic stress) and clinician impairment score (CGI) (Table 2). Subjects with complex trauma histories had more positive factors in MMPI-A Structural Summary (Immaturity, Health Concerns, Familial Alienation, Psychoticism) than other two groups; they scored higher on some MMPI-A clinical scale and Harris-Lingoes subscales, too (listed in Table 2). There were no statistical significant difference in total IQ in the three groups. On CBCL Scale Scores, there was only difference in ”Internalizing problems,” higher in the ”No trauma group” (Table 2).

**Table 2:** Psychometric measures of the three groups; differences were assessed by Chi-Square (^∗^) and One-Way ANOVA (°).

Measures N (%)	No trauma 59 (55.2)	Single trauma 12 (11.2)	Complex trauma 36 (33.6)	*p*-value
**Clinical Evaluation, Mean (SD)**				
CGI score	4.14 (0.69)	4.42 (0.79)	4.64 (0.93)	**0.014**
**Wisc-IV Quotient and indexes, Mean (SD)**				
Total IQ	99.80 (16.71)	94.22 (16.91)	91.38 (15.39)	0.062
Verbal Comprehension Index	102.67 (15.34)	99.23 (12.27)	94.94 (13.72)	0.060
Perceptual Reasoning Index	105.35 (16.91)	96.22 (17.09)	99.30 (14.64)	0.121
Working Memory Index	91.92 (15.99)	88.38 (15.97)	87.31 (14.88)	0.406
Processing Speed Index	97.48 (14.91)	93.25 (18.34)	89.34 (19.04)	0.100
**MMPI-A Structural summary, N (%)^∗^§**				
Positive presentation	3 (5.5)	0 (0.0)	2 (6.5)	0.745
Negative presentation	1 (1.8)	2 (28.6)	7 (22.6)	**0.003**
General Maladjustment	27 (49.1)	4 (57.1)	22 (71.0)	0.054
Immaturity	19 (34.5)	4 (57.1)	22 (71.0)	**0.005**
Disinhibition/Excitatory potential	13 (23.6)	3 (42.9)	12 (38.7)	0.256
Social discomfort	21 (38.2)	2 (28.6)	9 (29.0)	0.654
Health concerns	27 (49.1)	3 (42.9)	22 (71.0)	**0.039**
Naivete	4 (7.3)	1 (14.3)	5 (16.1)	0.423
Familial alienation	28 (50.9)	4 (57.1)	25 (80.6)	**0.024**
Psychoticism	26 (47.3)	3 (42.9)	25 (80.6)	**0.007**
**MMPI-A Basic clinical scale scores, Mean (SD)**				
F scale	58.82 (14.54)	65.00 (24.33)	70.84 (20.19)	**0.011**
Hysteria (3)	60.00 (10.95)	57.43 (12.57)	68.71 (12.06)	**0.002**
Psychopathic deviate (4)	59.13 (10.69)	61.00 (13.69)	69.06 (9.76)	** < 0.001**
Paranoia (6)	60.67 (12.42)	57.00 (13.08)	67.29 (11.34)	**0.027**
Schizophrenia (8)	58.73 (12.69)	65.14 (14.58)	67.35 (12.25)	**0.010**
**MMPI-A Harris-lingoes subscales, Mean (SD)**				
Lassitude-malaise (Hy3)	60.56 (11.28)	55.71 (13.20)	65.42 (9.36)	**0.046**
Self-alienation (Pd5)	57.76 (11.10)	60.43 (12.59)	64.81 (8.85)	**0.014**
Emotional alienation (Sc2)	57.25 (13.44)	64.71 (14.38)	66.00 (15.16)	**0.019**
Lack of ego mastery-cognitive (Sc3)	60.00 (14.05)	65.57 (15.35)	69.52 (11.39)	**0.008**
Lack of ego mastery-defective inhibition (Sc5)	54.67 (12.79)	63.29 (13.68)	61.39 (12.00)	**0.006**
**TSCC-A Scale scores, Mean (SD)**				
Anxiety	54.30 (14.91)	54.58 (16.43)	62.57 (14.97)	**0.010**
Depression	56.40 (15.73)	56.17 (13.82)	62.83 (11.54)	**0.030**
Anger	50.46 (10.61)	51.08 (9.26)	56.06 (12.77)	**0.020**
Post-traumatic stress	54.75 (13.76)	52.08 (12.41)	60.51 (16.62)	**0.040**
Dissociation	55.14 (13.65)	53.92 (11.70)	57.43 (14.59)	0.382
**CBCL Scale scores, Mean (SD)**				
Internalizing problems	69.05 (7.98)	61.00 (8.96)	66.63 (9.26)	**0.036**
Externalizing problems	61.32 (9.10)	60.50 (11.74)	62.90 (13.59)	0.768
Total problems	65.51 (8.44)	63.00 (8.85)	68.30 (10.75)	0.250

*Reported data are number of patients, with percentages shown in brackets, unless specified otherwise. ^§^ Prevalence of positive factors in the MMPI-A Structural Summary (>50% of the scales or subscales within a particular factor were at a critical level). Bolded values were statistically significant p-values.*

In multivariate logistic regression analyses, the complex trauma group was compared versus the single trauma and no trauma group (Table 3). Gender, age and affective disorders were generally unrelated to subjects’, clinicians’, and parents’ scores. About subjects’ self-assessment (MMPI-A Structural Summary Factors), complex trauma history was a statistically significant contributor to high scores on the Immaturity, Health Concerns, Familial Alienation and Psychoticism Factors, followed by presence of dissociative symptoms (except for Familial Alienation factor). About Health Concerns Factor, presence of neglect history or personality disorder diagnosis were related to lower scores.

**Table 3:** Variables associated with MMPI-A Factors and CGI-S scores in multivariate logistic linear regression.

	Yes	No	Multivariate p	OR	95% CI
**MMPI-A health concerns**	
Complex trauma	22 (71.0)	30 (48.4)	0.026	30.46	1.50–61.72
Dissociative symptoms	18 (69.2)	34 (50.7)	0.016	7.39	1.45–37.56
Personality disorder	11 (47.8)	41 (58.6)	0.030	0.153	0.028–0.836
Neglect	8 (44.4)	44 (58.7)	0.007	0.020	0.001–0.350
**MMPI-A immaturity**	
Complex trauma	22 (71.0)	23 (37.1)	0.005	92.22	3.93–216.54
Dissociative symptoms	20 (76.9)	25 (37.3)	0.003	12.01	2.35–51.33
**MMPI-A familial alienation**	
Complex trauma	25 (80.6)	32 (51.6)	0.038	13.48	1.15–117.43
**MMPI-A psychoticism**	
Complex trauma	25 (80.6)	29 (46.8)	0.009	155.84	3.55–383.44
Dissociative symptoms	23 (88.5)	31 (46.3)	0.006	12.33	2.03–64.79
**CGI-S score (>4)**	
Dissociative symptoms	21 (70.0)	17 (22.7)	0.031	3.47	1.12–10.78
Personality disorder	14 (58.3)	24 (29.6)	0.033	3.96	1.12–14.06
Psychotic disorder	21 (20.0)	17 (22.7)	0.027	6.06	1.23–29.79
**CBCL problem total score**	
*No statistical significance.*					

*For every variable the same independent variables were considered: Male gender, Age, Complex trauma history, PTSD diagnosis and Complex trauma history, Sexual abuse history, Physical abuse history, Neglect history, Presence of dissociative symptoms, Psychotic disorder diagnosis, Personality disorder diagnosis, Affective disorder diagnosis. Only statistical significant variables were reported in the table. Reported data are number of patients, with percentages shown in brackets, unless specified otherwise.*

Complex trauma history was not a significant contributor in CGI-S score (>4); presence of dissociative symptoms, personality and psychotic disorder diagnosis was related to higher clinician impairment scores (Table 3).

No significant contributors were identified on CBCL Total, Internalizing and Externalizing Problems clinical range scores (T > 64) (Table 3).

## Discussion

The type of trauma (sexual abuse, mistreatment, neglect) does not appear to significantly affect psychiatric diagnosis, clinical severity (expressed by the score to CGI-S), nor on the type or intensity of symptoms reported by patients or by caregiver, with one exception. Patients with a history of neglect report less somatic concerns (as stated in MMPI-A questionnaire) than individuals who have not experienced this kind of experience. That finding appears difficult to interpret, even because individuals with complex trauma, by definition, experience different types of trauma. In our sample we have only three subjects with a history of neglect, without other types of trauma; therefore, there was not possible a statistical comparison to confirm this data in the absence of other types of traumatic events.

In literature there are several studies that have tried to find a correspondence between type of trauma, symptoms and course, with different and sometimes apparently contradictory results; several Authors report how very often children are simultaneously or in quick succession subjected to different types of traumatic events and therefore the predictor factors of outcomes are the severity, the length of exposure and the age of the child, rather than the sexual, physical or psychological nature of the event (English et al., 2005; Jonkman et al., 2013; Mueller-Pfeiffer et al., 2013; Bonvanie et al., 2015; Vachon et al., 2015; Hodgdon et al., 2018).

The overall rate of PTSD was estimated in 15.9% in traumatized children and adolescents, which varied according to the kind of trauma and gender; almost one in four who experienced interpersonal trauma, developed PTSD (Alisic et al., 2018). In Single Trauma group we found a smaller rate of PTSD (8.3%); we found a much higher rate of PTSD (58.3%) in Complex Trauma group, probably because complex trauma is more often chronic, erodes social support, leads to more sense of fault or other maladaptive cognitions, represents a disloyalty, in ways that affect daily functioning. Except for PTSD diagnosis, there were no differences in psychiatric diagnosis between the three groups, but Complex Trauma Group had higher Clinician Impairment scores. Clinicians rated more impairment in patients with personality or psychotic disorders; although complex trauma history status *per se* was not correlated with impairment severity, the presence of dissociative symptoms was associated to the high scores on CGI-S.

In our sample, both dissociative symptomatology and complex trauma history were significantly related to MMPI-A Immaturity and Psychoticism Factors (Immaturity, Psychoticism, and Health Concerns), too. Adolescents who often were described as selfish, narcissistic, impulsive, and limited in terms of self-awareness or psychological insight get high scores on the Immaturity Factor. They more frequently manifest behavioral problems in the school setting and have difficulties in relationships or are litigious. They could have poor peer relationships characterized by bullying, physical aggression and threats. Adolescents who get significant elevations on the Psychoticism Factor are often socially disengaged and have higher likelihood of being teased or refused by peers. They are likely to manifest persecutory ideas, bizarre sensory experiences, and difficulty in the emotional self-regulation, with possibility of tantrums (Archer, 2005).

Dissociative symptoms are more frequently observed in individuals with histories of complex trauma, who suffer from post-traumatic stress symptoms and affect dysregulation (van der Kolk et al., 2005; Briere, 2006; Mueller-Pfeiffer et al., 2013).

Although dissociative symptoms are a defense mechanism against stress associated with traumatic events, they also may arise as a way to diminish the emotional responses associated with triggered traumatic memories, even after a long period from the traumatic event. Thus, thoughts are disconnected from emotions, there is a lack of awareness on the somatic feelings and some behaviors can become repetitive and automatic, without being a planning or a complete awareness.

Therefore, dissociation may on one side expose the child to other traumatic experiences (for example accidents), on the other side it can interfere with the relationships, as it has a negative impact on emotional self-regulation and attachment (Dell and O’Neil, 2009).

Complex trauma history and dissociative symptoms related to high scores on Health Concerns Factor, too. High scores on this factor are obtained by adolescents who often display fatigability and limited endurance, sleeping difficulties, somatic complaints and the perception of poor physical health. They could have patterns of overreaction to stress, often involving development of physical symptoms, and little insight into problem areas (Archer, 2005).

Somatic symptoms (for example abdominal pain, headache, pain in the limbs) are very frequent in childhood, also in non-clinical samples; nevertheless, traumatized children show them with a higher frequency and intensity (Bonvanie et al., 2015). Such symptoms can significantly interfere with the quality of life, the individual functioning and school attendance (Kugler et al., 2012). Seng et al. (2005) found that PTSD is associated with adverse health outcomes even in adolescence; they also observed that when PTSD is comorbid with dissociative symptoms, depression or borderline personality disorders, there was more probability to have physical comorbidity.

The dissociation has also been related to some alterations in the brain (decreased left hippocampal volume) and in cerebrospinal fluid levels of neurotransmitters and their metabolites (Cook et al., 2005; Keding and Herringa, 2015). Diseth (2005) has reported that the cerebral basis and mechanisms for the trauma-related dissociation observed in PTSD, are common to dissociative and somatic disorders; it is therefore conceivable that PTSD and somatic symptom disorders may be specific forms of trauma-related dissociative processes. This is a fascinating hypothesis that requires further evidences and confirmations.

Adolescents with complex trauma history produced also significant elevations on the Familial Alienation Factor; they are more likely to go through significant family problems and to come upon disciplinary problems at school (Archer, 2005). They are often described as aggressive (verbally or physically), disobedient, hostile, especially in domestic situations, probably because we defined “complex trauma” as “multiple experiences of emotional, physical, or sexual abuse and emotional or physical neglect at an early age within a caregiving context.” When the trauma originates in the contest of the relationship with the caregiver the system of attachment can be severely damaged. Posttraumatic impairment of neurobiological integrity and the impairment of the attachment system can lead to severe problems with the affective and behavioral regulation: the behavior for example can be strictly controlled or otherwise dysregulated with aggressive, provocative or oppositional acts (Cook et al., 2005; van der Kolk et al., 2005).

Our study has many advantages, but also several limitations. We used standardized tools for the assessment and we considered as traumatized only those subjects with a documented history about it, therefore, we didn’t rely on patient’s statements.

Although on one side this represents an advantage, on the other side we must give consideration to the possibility that patients in the other groups have undergone traumatic experiences that aren’t so far emerged. We enrolled only subjects within 12 and 18 years at a secondary center; those subjects presented symptoms of such intensity to require a diagnostic evaluation and a treatment. The three groups had small size and findings might not be generalizable to people with traumatic histories in other age groups or in other settings, for example to people who don’t have a diagnosis, who have symptomps of low intensity or that don’t require a neuropsychiatric evaluation or treatment.

We considered only certain types of interpersonal trauma; in our sample there are subjects exposed to accidents, natural disasters (such as earthquakes), sudden death of points of reference, real danger of life, psychological violence. The study is retrospective than the traumatic event and there are no data from standardized tests that describe the functioning of the subjects in the period prior to trauma; furthermore, individuals with complex trauma are placed in communities or in foster families, therefore, even the clinical history can be reconstructed only indirectly. It is often difficult to establish when traumatic experiences began.

It is, therefore, possible that some symptom manifestations and malaise are previous than the trauma. In spite of these limitations, we believe that our study strengthens available evidence that in trauma-exposed adolescents, the short and long-term effects of complex traumatic events can lead to other psychiatric diagnosis than PTSD, (such as affective, personality, and psychotic disorders), and may cause dissociative and somatic symptomatology, which may be more disabling than post-traumatic symptoms (Jonkman et al., 2013; Vachon et al., 2015; Yearwood et al., 2017).

Until now, no unique correspondence has yet been found between the type of trauma and its consequences, topic on which other studies are needed; therefore, an analyzed and appropriate assessment of patients with a complex traumatic history appears necessary and indispensable, in order to define the full burden of trauma and to implement appropriate and individualized therapeutic interventions.

## Ethics Statement

This protectstudy was carried out in accordance with the recommendations of the local Ethics Committee “Comitato Etico dell’Insubria.” The protocol was approved by the local Ethics Committee “Comitato Etico dell’Insubria.” All subjects gave written informed consent in accordance with the Declaration of Helsinki.

## Author Contributions

CL conceived the research, prepared the materials, analyzed the data, and drafted the manuscript. CL and SC collected the data. MA, SC, GR, and CT contributed to the theoretical rationale for the study and the research design. All authors contributed to the interpretation of data, critically revised, and approved the manuscript.

## Conflict of Interest Statement

The authors declare that the research was conducted in the absence of any commercial or financial relationships that could be construed as a potential conflict of interest.
